# Spatial and temporal specificity of Ca^2+^ signalling in *Chlamydomonas reinhardtii* in response to osmotic stress

**DOI:** 10.1111/nph.14128

**Published:** 2016-08-12

**Authors:** Peter Bickerton, Simone Sello, Colin Brownlee, Jon K. Pittman, Glen L. Wheeler

**Affiliations:** ^1^ Marine Biological AssociationCitadel HillPlymouthPL1 2PBUK; ^2^ Faculty of Life SciencesUniversity of ManchesterOxford RoadManchesterM13 9PTUK; ^3^ Department of BiologyUniversity of PadovaVia U. Bassi 58/B35131PadovaItaly; ^4^ School of Ocean and Earth ScienceUniversity of SouthamptonSouthamptonSO14 3ZHUK

**Keywords:** calcium, *Chlamydomonas reinhardtii*, flagella, green algae, osmotic stress, signalling

## Abstract

- Ca^2+^‐dependent signalling processes enable plants to perceive and respond to diverse environmental stressors, such as osmotic stress. A clear understanding of the role of spatiotemporal Ca^2+^ signalling in green algal lineages is necessary in order to understand how the Ca^2+^ signalling machinery has evolved in land plants.
- We used single‐cell imaging of Ca^2+^‐responsive fluorescent dyes in the unicellular green alga *Chlamydomonas reinhardtii* to examine the specificity of spatial and temporal dynamics of Ca^2+^ elevations in the cytosol and flagella in response to salinity and osmotic stress.
- We found that salt stress induced a single Ca^2+^ elevation that was modulated by the strength of the stimulus and originated in the apex of the cell, spreading as a fast Ca^2+^ wave. By contrast, hypo‐osmotic stress induced a series of repetitive Ca^2+^ elevations in the cytosol that were spatially uniform. Hypo‐osmotic stimuli also induced Ca^2+^ elevations in the flagella that occurred independently from those in the cytosol.
- Our results indicate that the requirement for Ca^2+^ signalling in response to osmotic stress is conserved between land plants and green algae, but the distinct spatial and temporal dynamics of osmotic Ca^2+^ elevations in *C. reinhardtii* suggest important mechanistic differences between the two lineages.

## Introduction

The ability of an organism to sense and respond to its environment is fundamental for its survival. In plants, calcium (Ca^2+^)‐dependent signalling processes play a central role in the response to many environmental stimuli, including mechanical stimulation, osmotic stress, oxidative stress and temperature shock (Knight *et al*., 1992, 1997; Takahashi *et al*., 1997; Kiegle *et al*., 2000; Pei *et al*., 2000). It is likely that the development of these signalling pathways helped plants to colonize land and become the dominant primary producers in terrestrial ecosystems. However, our ability to understand how and why these important signalling processes have evolved in plants is limited, as the role of Ca^2+^ signalling in response to environmental stimuli in the green algae (Chlorophyta), which represent the other major lineage in the Viridiplantae, is not well characterized.

Many of the diverse environmental stimuli encountered by plants give rise to activation of Ca^2+^‐permeable channels in the plasma membrane or internal membranes, leading to transient elevations in cytosolic Ca^2+^ ([Ca^2+^]_cyt_) which are sensed by a range of downstream effector proteins (such as calmodulin (CaM), calmodulin‐like proteins (CMLs), the calcium‐dependent protein kinases (CDPKs) or the calcineurin B‐like (CBL) calcium sensor proteins and their CBL‐interacting protein kinases (CIPKs)) (Hetherington & Brownlee, 2004; Edel & Kudla, 2015). As [Ca^2+^]_cyt_ elevations are generated by a wide variety of stimuli, the spatial and temporal dynamics of each [Ca^2+^]_cyt_ elevation are important in conveying specificity (McAinsh & Pittman, 2009; Whalley *et al*., 2011). For example, plant [Ca^2+^]_cyt_ elevations can take the form of brief spikes, extended elevations or a series of oscillations. In combination with the broad range of downstream responders, the distinct spatiotemporal dynamics of [Ca^2+^]_cyt_ elevations enable the cell to use Ca^2+^ signalling in response to many different stimuli.

Vascular plants lack clear homologues of several important classes of Ca^2+^ channel found in animal cells, such as the four‐domain voltage‐dependent Ca^2+^ channels (VDCCs), the transient receptor potential (TRP) channels and the inositol triphosphate receptor (IP_3_R) (Wheeler & Brownlee, 2008; Verret *et al*., 2010; Edel & Kudla, 2015). However, these channels are present in green algal genomes (Merchant *et al*., 2007; Wheeler & Brownlee, 2008), suggesting that there may be important differences in the signalling mechanisms between plants and green algae. While [Ca^2+^]_cyt_ elevations in plants have been extensively characterized (McAinsh & Pittman, 2009), direct observations of [Ca^2+^]_cyt_ elevations in green algae remain limited. [Ca^2+^]_cyt_ elevations have been observed in response to ‘light‐off’ stimuli in the freshwater alga *Eremosphaera viridis* (Bauer *et al*., 1997) and during the settlement of *Ulva linza* zoospores (Thompson *et al*., 2007). [Ca^2+^]_cyt_ elevations relating to the deflagellation process have been extensively characterized in the freshwater alga *Chlamydomonas reinhardtii* (Wheeler *et al*., 2008). *Chlamydomonas reinhardtii* can excise its flagella via a Ca^2+^‐dependent signalling pathway in response to various stressors, including osmotic stress (Quarmby, 1996; Meijer *et al*., 2002). The addition of 20 mM external Ca^2+^ to *C. reinhardtii* led to a series of repetitive [Ca^2+^]_cyt_ elevations that were linked to the process of flagellar excision (Wheeler *et al*., 2008). The rapid and dynamic nature of these [Ca^2+^]_cyt_ elevations in *C. reinhardtii* was considerably different from those observed in vascular plants, where [Ca^2+^]_cyt_ transients commonly last for many seconds. Repetitive [Ca^2+^]_cyt_ spiking in vascular plants also generally occurs over timescales of minutes rather than seconds (e.g. Ca^2+^ spiking in root hairs induced by nodulation factors) (Wais *et al*., 2000).

*Chlamydomonas reinhardtii* is a motile organism, which may account for the dynamic nature of some of the signalling processes within the cell. The motile responses of swimming *C. reinhardtii* cells to light have been well documented and are mediated by changes in flagellar Ca^2+^ ([Ca^2+^]_fla_). Voltage‐gated Ca^2+^ channels in the flagella are activated by a whole‐cell depolarization mediated by channelrhodopsin, a light‐gated ion channel situated in the plasma membrane adjacent to the eyespot (Harz & Hegemann, 1991; Fujiu *et al*., 2009). In addition to swimming, *C. reinhardtii* cells can move by adhering to a substrate via their flagella and gliding along that surface (Bloodgood, 1981). Gliding motility is mediated by the movement of adherent glycoproteins in the flagellar membrane, which are driven along the length of the flagellar axoneme by the microtubule motors responsible for intraflagellar transport (IFT) (Collingridge *et al*., 2013; Shih *et al*., 2013). We have previously demonstrated that the movement of IFT proteins during gliding motility is regulated by flagellar Ca^2+^ signalling (Collingridge *et al*., 2013). The flagella of gliding *C. reinhardtii* cells are arranged at 180° to each other and gliding motility initiates when the pulling force in one flagellum overcomes the resistance of the other (Bloodgood, 1981). Direct observation of flagellar Ca^2+^ ([Ca^2+^]_fla_) in gliding *C. reinhardtii* cells indicates the potential for complex and highly dynamic signalling, with each individual flagellum capable of generating very rapid, repetitive [Ca^2+^]_fla_ elevations independently of the cytosol and the other flagellum (Collingridge *et al*., 2013).

While the role of Ca^2+^ signalling in flagellar‐related processes in *C. reinhardtii* is well established, its role in regulating processes in the cell body has been less thoroughly explored. Recent progress indicates a role for Ca^2+^ in the cellular responses to nutrient starvation (Motiwalla *et al*., 2014) and in regulating photoacclimation, through the activity of the chloroplast‐localized calcium sensor protein (CAS) (Petroutsos *et al*., 2011). However, the dynamics of [Ca^2+^]_cyt_ elevations in response to these and other environmental stimuli in *C. reinhardtii* have not been characterized.

In order to gain a better understanding of the role of Ca^2+^ signalling in green algae, we performed a detailed examination of the dynamics of [Ca^2+^]_cyt_ elevations generated by *C. reinhardtii* cells in response to osmotic stress. We found that salinity stress and hypo‐osmotic stimuli induced [Ca^2+^]_cyt_ elevations with distinct spatial and temporal characteristics. Hypo‐osmotic stress also induced repetitive [Ca^2+^]_fla_ elevations that were independent of [Ca^2+^]_cyt_, indicating that the flagella, although continuous with the cytosol, can act as distinct Ca^2+^ signalling compartments in response to environmental stimuli.

## Materials and Methods

### Algal strains and growth conditions

*Chlamydomonas reinhardtii* Dangeard strains CC1021 mt+ (wild type) and *cw15* (CCAP 11/32 CW15+, cell wall deficient) were obtained from the Chlamydomonas Resource Center (University of Minnesota, St Paul, MN, USA) and the Culture Collection of Algae and Protozoa (Scottish Association for Marine Science, Oban, UK), respectively. The *ift20* IFT20‐mCherry strain was a gift from Karl Lechtreck (Lechtreck *et al*., 2009). Cultures were grown in standard Tris‐acetate‐phosphate (TAP) liquid medium at 23°C with a 16 h : 8 h, light : dark cycle and a light intensity of 100 μmol m^−2^ s^−1^.

### Biolistic loading of dextran‐conjugated fluorescent dyes

Cells were simultaneously loaded with two fluorescent dyes, the Ca^2+^‐responsive dye Oregon Green‐BAPTA Dextran (OG) (10 000 MW) and the reference dye Texas Red Dextran (TR) (10 000 MW) (Invitrogen Ltd, Paisley, UK). Dextran‐conjugated dyes were used to avoid the problems with dye compartmentalization commonly found with plant and algal cells (Bothwell *et al*., 2006). OG was used for this study rather than Fluo‐4 dextran used previously (Wheeler *et al*., 2008) as OG has a much greater fluorescence than Fluo‐4 when in the Ca^2+^‐unbound state, which reduces technical issues associated with chlorophyll autofluorescence and also aids imaging of the flagella by Total Internal Reflection Fluorescence (TIRF) microscopy. *Chlamydomonas reinhardtii* cells were concentrated by centrifugation (400 ***g*** for 5 min) and washed with biolistic loading buffer (BLB) (10 mM HEPES, pH 7.4, 20 μM K^+^ glutamate and 50 mM sorbitol), and then biolistic loading was performed using a Bio‐Rad PDS‐1000 delivery system with 1100‐psi rupture discs, as described previously (Wheeler *et al*., 2008). Samples were washed and resuspended in TAP medium and left for 2 h to recover under normal growth conditions. After this period, loaded cells were chosen that were able to swim and glide normally, indicating that they were fully recovered from the loading process. We confirmed that the biolistically loaded fluorescent dyes were correctly localized to the cytosol of *C. reinhardtii*, using confocal laser microscopy (Zeiss LSM510; excitation 488 nm; emission 500–530 and 650–710 nm). The dyes demonstrated a homogenous cytosolic localization, with no evidence of compartmentalization. Chlorophyll autofluorescence was negligible relative to OG, allowing us to subsequently use epifluorescent microscopy to image [Ca^2+^]_cyt_.

### Ca^2+^ imaging

Before imaging, cells were resuspended in *Chlamydomonas* Assay Buffer (CAB) containing 5 mM HEPES, 1 mM HCl, 1 mM KCl, 0.2 mM Ethylene glycol‐bis(2‐aminoethylether)‐N,N,N′,N′‐tetraacetic acid and 0.5 mM CaCl_2_ with pH adjusted to 7.4 using N‐methyl‐d‐glucamine (NMDG). Free Ca^2+^ was calculated to be 301 μM using Maxchelator (http://maxchelator.stanford.edu/). Cells were placed in 35‐mm glass‐bottomed dishes (In Vitro Scientific, Sunnyvale, CA, USA) coated with 0.01% poly‐L‐lysine (Sigma‐Aldrich, St Louis, MO, USA) to encourage adherence of the cells. Cells were perfused with CAB buffer at a flow rate of 3 ml min^−1^ during imaging.

Cells were imaged by epifluorescence microscopy at room temperature using a Nikon Eclipse Ti with a ×100, 1.49 NA oil immersion objective and detection with a Photometrics Evolve EM‐CCD camera (Photometrics, Tucson, AZ, USA). Simultaneous excitation of OG and TR was performed using 488‐ and 561‐nm lasers (Coherent, Santa Clara, CA, USA). A Dual‐View beam splitter device (Photometrics, Tucson, AZ, USA) was used to detect emission at 500–550 and 575–625 nm, respectively. Images were captured using nis‐elements v.3.1 software (Nikon, Japan) with a 200–300‐ms exposure (frame rate of 3.33–5 frames s^−1^). The same microscopy equipment was used in TIRF mode for flagellar imaging. IFT20‐mCherry was visualized in TIRF mode using 561‐nm laser excitation and 575–625‐nm emission.

NaCl shocks were delivered by rapidly switching the perfusion from CAB to CAB + 50–400 mM NaCl. Hypo‐osmotic treatments were delivered by switching the perfusion to deionized water containing 50 μM CaCl_2_. To investigate the response to hypo‐osmotic shock in the absence of contractile vacuole activity, cells were equilibrated to CAB containing 100 mM sucrose for at least 1 h. In order to assess whether extracellular Ca^2+^ is involved in [Ca^2+^]_cyt_ elevations in response to osmotic shock, cells were initially perfused with Ca^2+^‐free CAB (containing 200 μM EGTA without added CaCl_2_) and a hypo‐osmotic shock was administered using deionized water containing 50 μM EGTA (adjusted to pH 7). In order to assess the involvement of mechanosensitive ion channels in the generation of [Ca^2+^]_cyt_ elevations*,* cells were pretreated for at least 10 min with a final concentration of either 5 μM GsMTx4 (Smartox Biotechnology, Saint‐Martin‐d'Hères, France) or 10 μM Ruthenium red (RuR) (Sigma‐Aldrich) before the perfusion with distilled water or distilled water containing 10 μM RuR, respectively. RuR did not exhibit any fluorescent properties that interfered with Ca^2+^ imaging.

### Data processing

Changes in [Ca^2+^]_cyt_ were identified by calculating the fluorescence intensity ratio between OG and TR for a defined region of interest within each *C. reinhardtii* cell. Ratio traces were smoothed using a Savitsky–Golay filter (window 5, order 2) and a baseline trace was created using an asymmetric least squares smoothing baseline algorithm (originpro 2016; OriginLab Corporation, Northampton, MA, USA) (Supporting Information Fig. S1). The smoothed OG/TR traces were divided by the baseline trace to determine the relative change in fluorescence. [Ca^2+^]_cyt_ elevations were defined as any increase in OG/TR fluorescence above a threshold value (3%) that persisted for more than one frame. We calculated that an OG/TR ≥ 3% required a minimum signal to noise ratio (SNR) of 10, with SNR defined as signal intensity/SD of noise. Contractile vacuole activity was detected by measuring changes in the fluorescence intensity of TR in a region of interest encompassing each contractile vacuole. For visualization of [Ca^2+^]_cyt_ dynamics in cells, imagej software was used to divide each OG image by a rolling median image from 20 frames to produce a pseudocoloured ratio image. Previous researchers have demonstrated that standard Ca^2+^ ionophores do not work well in *C. reinhardtii* (Braun & Hegemann, 1999), which precludes an *in vivo* calibration of OG. We therefore performed an *in vitro* calibration of this dye using Calcium Calibration Buffer Kit no. 1 (Life Technologies Ltd, Paisley, UK), allowing the increase in [Ca^2+^]_cyt_ for a given change in fluorescence to be estimated, assuming a resting [Ca^2+^]_cyt_ of 100 nM (Fig. S2). Data obtained from all treatments are shown as mean ± SE.

## Results

### Salt stress induces a single [Ca^2+^]_cyt_ elevation in *Chlamydomonas reinhardtii*

**Figure 1:**
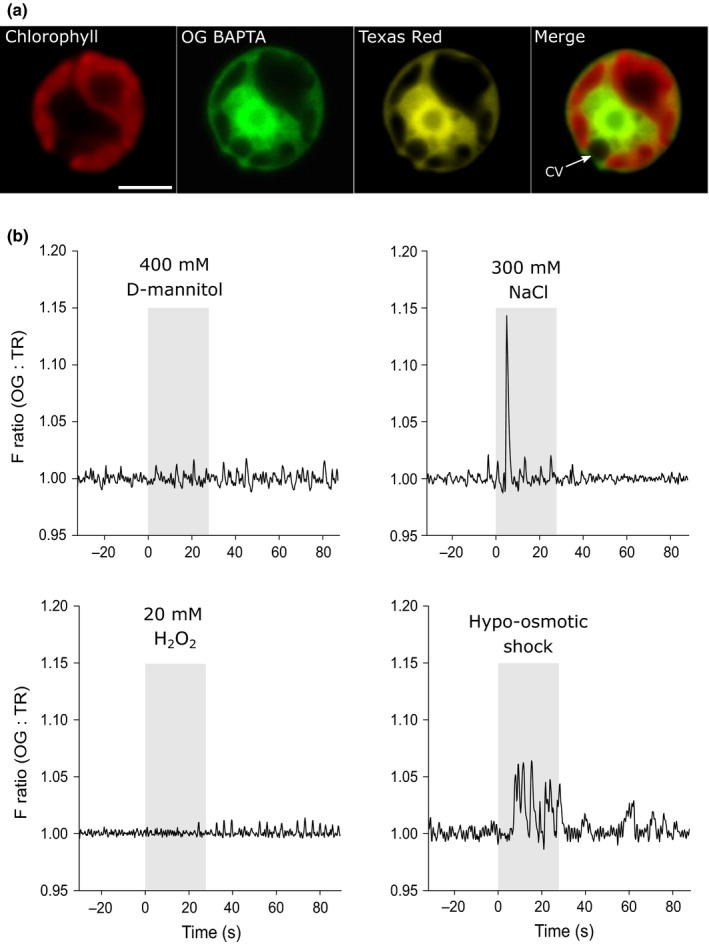
Osmotic shock induces [Ca^2+^]_cyt_ elevations in *Chlamydomonas reinhardtii*. (a) Confocal laser microscopy image of a CC1021 *C. reinhardtii* cell demonstrating that the biolistically loaded Ca^2+^‐responsive fluorescent dye Oregon Green BAPTA dextran (OG) is correctly localized to the cytosol. The localization of the reference dye Texas Red dextran (TR) is also shown, along with chlorophyll autofluorescence. The position of a contractile vacuole (CV) is marked. Bar, 5 μm. (b) Ca^2+^ signalling viewed by epifluorescent microscopy in response to four different stimuli applied to cells for 30 s. No [Ca^2+^]_cyt_ elevations were observed following the addition of 400 mM mannitol (*n *=* *18) or 20 mM hydrogen peroxide (H_2_O_2_) (*n *=* *18), but there were distinct [Ca^2+^]_cyt_ elevations in response to 300 mM NaCl (*n *=* *9 out of 61 cells) or to hypo‐osmotic shock (induced by deionized water) (*n *=* *22 out of 31 cells). F ratio denotes fluorescence ratio of OG/TR.

In order to examine whether Ca^2+^ signalling processes are conserved between vascular plants and green algae, we applied four external stimuli that routinely induce [Ca^2+^]_cyt_ elevations in plants to *C*. *reinhardtii* strain CC1201. We found that hydrogen peroxide (up to 20 mM) and mannitol (up to 400 mM) did not induce [Ca^2+^]_cyt_ elevations in *C. reinhardtii* (Fig. 1a,b). However, NaCl and hypo‐osmotic shock resulted in [Ca^2+^]_cyt_ elevations and these stimuli were therefore examined in greater detail. We examined Ca^2+^ signalling in response to salt stress in two *C. reinhardtii* strains, the CC1021 wild‐type strain and *cw15*, a cell wall deficient mutant. Application of 300 mM NaCl to CC1021 resulted in [Ca^2+^]_cyt_ elevations in nine out of 61 cells. These cells exhibited a single rapid [Ca^2+^]_cyt_ elevation lasting for 2.04 ± 0.33 s that occurred 7.04 ± 0.73 s after the stimulus was applied. The mean maximal amplitude of the OG/TR increase was 8.93 ± 0.97%, which equates to a mean maximal [Ca^2+^]_cyt_ of 184 nM, assuming a resting [Ca^2+^]_cyt_ of 100 nM. The nature of the [Ca^2+^]_cyt_ elevations was highly consistent, although it is clear that only a small proportion of the cells exhibit this response (14.8%), suggesting that a threshold stimulus is required to trigger a [Ca^2+^]_cyt_ elevation. Very few [Ca^2+^]_cyt_ elevations were observed at lower concentrations of NaCl (no cells responded at 150 mM (*n *=* *11) and only one out of 12 cells at 200 mM NaCl). Higher concentrations of NaCl (400 mM) shrank the cells excessively, preventing accurate determination of [Ca^2+^]_cyt_. Previous researchers have demonstrated that Ca^2+^‐dependent signalling pathways in *C. reinhardtii* can be influenced by the concentration of external Ca^2+^ in the surrounding media (Quarmby & Hartzell, 1994; Petroutsos *et al*., 2011). However, we found that CC1021 cells acclimated to 10 mM external Ca^2+^ showed a high frequency of spontaneous [Ca^2+^]_cyt_ elevations, making it difficult to examine their response to NaCl (Fig. S3).

**Figure 2:**
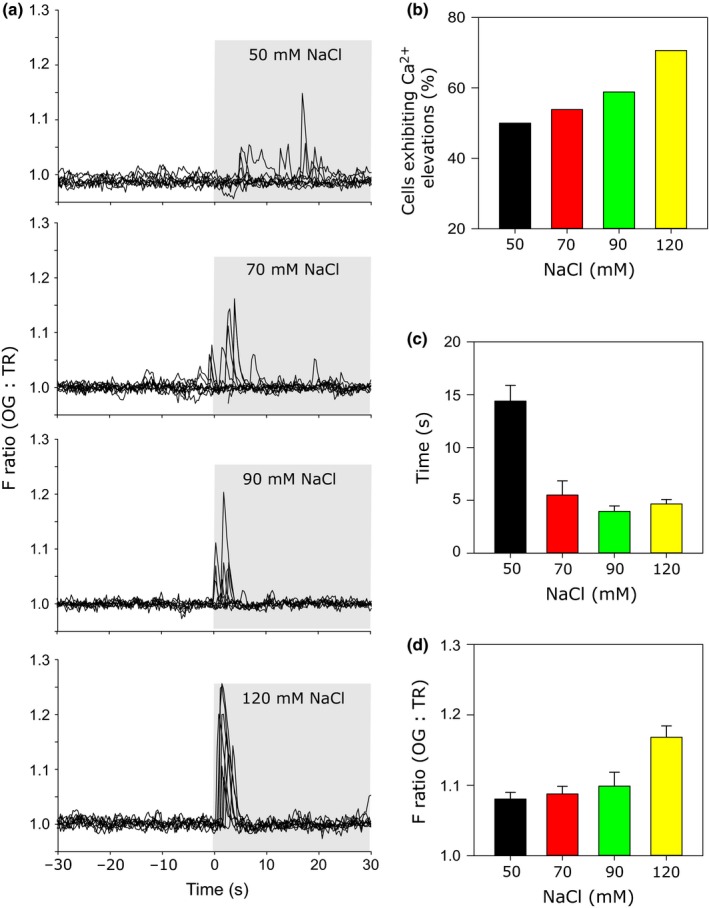
Stimulus‐specific signalling in response to NaCl. (a) Characterization of [Ca^2+^]_cyt_ elevations induced by different NaCl concentrations in cell wall‐deficient *Chlamydomonas reinhardtii cw15* cells. The Oregon Green BAPTA dextran (OG) : Texas Red dextran (TR) fluorescence (F) ratios from eight representative cells are shown for each concentration. The NaCl stress was applied at 30 s. It is clear that the timing and amplitude of the [Ca^2+^]_cyt_ elevations change with increasing NaCl concentrations. (b) The percentage of cells exhibiting significant [Ca^2+^]_cyt_ elevations in response to NaCl (50 mM, *n *=* *14; 70 mM, *n *=* *14; 90 mM, *n *=* *10; 120 mM, *n *=* *12). (c) Mean time after the application of NaCl that the initial [Ca^2+^]_cyt_ elevation was observed. (d) Mean maximal amplitude of the [Ca^2+^]_cyt_ elevations in *cw15* cells treated with NaCl. Error bars denote SE.

Ca^2+^ signalling in the cell wall‐deficient strain *cw15* was significantly more sensitive to salinity stress than in CC1021. We found that 50 mM NaCl induced a single [Ca^2+^]_cyt_ elevation in 55% of *cw15* cells (*n *=* *14), with a mean maximal increase in the OG : TR ratio of 8.0 ± 0.9% (176 nM [Ca^2+^]_cyt_) which occurred 14.4 ± 1.5 s after the application of the stimulus (Fig. 2). Treatment of *cw15* cells with higher concentrations of NaCl (70, 90 and 120 mM) resulted in progressive increases in the mean maximal amplitude of the [Ca^2+^]_cyt_ elevations and in the proportion of cells exhibiting [Ca^2+^]_cyt_ elevations (Fig. 2). The delay between the stimulus and the response also decreased. For example, 120 mM NaCl resulted in single [Ca^2+^]_cyt_ elevations in over 80% of cells. These [Ca^2+^]_cyt_ elevations had a mean maximal amplitude of 16.8 ± 1.6% (equating to a [Ca^2+^]_cyt_ of 261 nM) and occurred 4.7 ± 0.4 s after the application of the stimulus. We conclude that the timing and amplitude of NaCl‐induced [Ca^2+^]_cyt_ elevations are sensitive to the intensity of the stimulus, which may contribute to specificity in downstream responses.

### NaCl‐induced [Ca^2+^]_cyt_ elevations propagate as a fast Ca^2+^ wave

**Figure 3:**
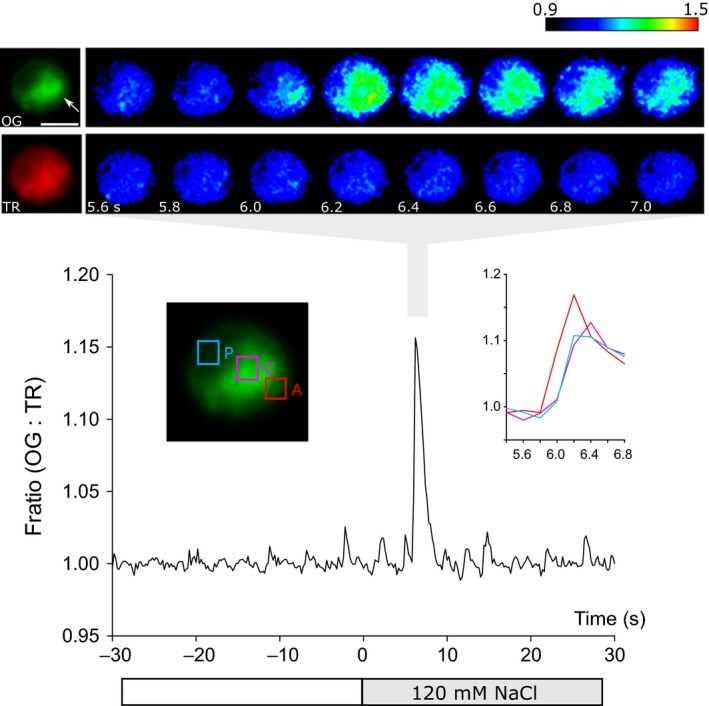
[Ca^2+^]_cyt_ elevations in response to NaCl initiate in the apical region. Spatial and temporal characterization of a [Ca^2+^]_cyt_ elevation induced by 300 mM NaCl in a *Chlamydomonas reinhardtii* CC1021 cell was carried out. The graph displays the change in fluorescence ratio (Oregon Green BAPTA dextran : Texas Red dextran (OG : TR)). The pseudocoloured images represent the change in fluorescence (Δ*F*/*F*_0_) in the OG and TR channels. The images show that the [Ca^2+^]_cyt_ elevation in response to NaCl initiates in the apical region (arrowed) and rapidly propagates to the rest of the cell (200 ms per frame). The inset shows an expanded timescale of the [Ca^2+^]_cyt_ elevation in apical, central and posterior regions of interest within the cell. The cell shown is representative of five CC1021 cells examined and similar spatial characteristics were observed in *cw15* cells (*n *=* *5). Bar, 5 μm.

We next examined the spatial characteristics of [Ca^2+^]_cyt_ elevations induced by NaCl. We found that the [Ca^2+^]_cyt_ elevations induced by 300 mM NaCl in CC1021 originated in the apex of the cell and rapidly spread to the remainder of the cell, representing a fast Ca^2+^ wave (*n *=* *5 cells examined where the apex of the cell could be clearly distinguished) (Fig. 3). [Ca^2+^]_cyt_ elevations induced by 120 mM NaCl in *cw15* also originated in the apical region and rapidly spread to the rest of the cell (*n *=* *5 cells, Video S1). *Chlamydomonas reinhardtii* is a small cell (5–10 μm), making it difficult to accurately determine the speed of the propagating Ca^2+^ wave. However, it was clear that the initial apical [Ca^2+^]_cyt_ elevation spread to the remainder of the cell within 0.5–1 s, representing a speed of *c*. 10–20 μm s^−1^. We have previously observed that 20 mM external Ca^2+^ can stimulate a Ca^2+^ wave that propagates from the apex of *C. reinhardtii* cells at a mean speed of 30 μm s^−1^ (Wheeler *et al*., 2008). These propagation speeds are similar to the fast Ca^2+^ waves in animal cells that are generated via the process of Ca^2+^‐induced Ca^2+^ release (CICR) from the endoplasmic reticulum (Jaffe, 2010). Our results also suggest that the apex of the *C. reinhardtii* cell plays an important role in sensing and responding to salt stress.

To examine whether the NaCl‐induced [Ca^2+^]_cyt_ elevations in *C. reinhardtii* were dependent on Ca^2+^ influx from the external medium, we applied 120 mM NaCl to *cw15* cells in the absence of external Ca^2+^ (i.e. CAB assay buffer containing 200 μM EGTA, but without added CaCl_2_). We did not observe [Ca^2+^]_cyt_ elevations in any cells (*n *=* *16). This indicates that an influx of external Ca^2+^ is required to initiate the [Ca^2+^]_cyt_ elevations induced by NaCl, although the [Ca^2+^]_cyt_ elevations may subsequently propagate through the release of Ca^2+^ from internal stores. The absolute dependence on external Ca^2+^ in *C. reinhardtii* contrasts with Arabidopsis, where NaCl‐induced [Ca^2+^]_cyt_ elevations were only partially repressed in the presence of La^3+^ or EGTA (Knight *et al*., 1997). NaCl‐induced [Ca^2+^]_cyt_ elevations in Arabidopsis are also partially inhibited by nicotinamide, an inhibitor of Ca^2+^ release from internal stores by cyclic‐ADP ribose (Abdul‐Awal *et al*., 2016).

### Hypo‐osmotic stress induces a series of repetitive [Ca^2+^]_cyt_ elevations

**Figure 4:**
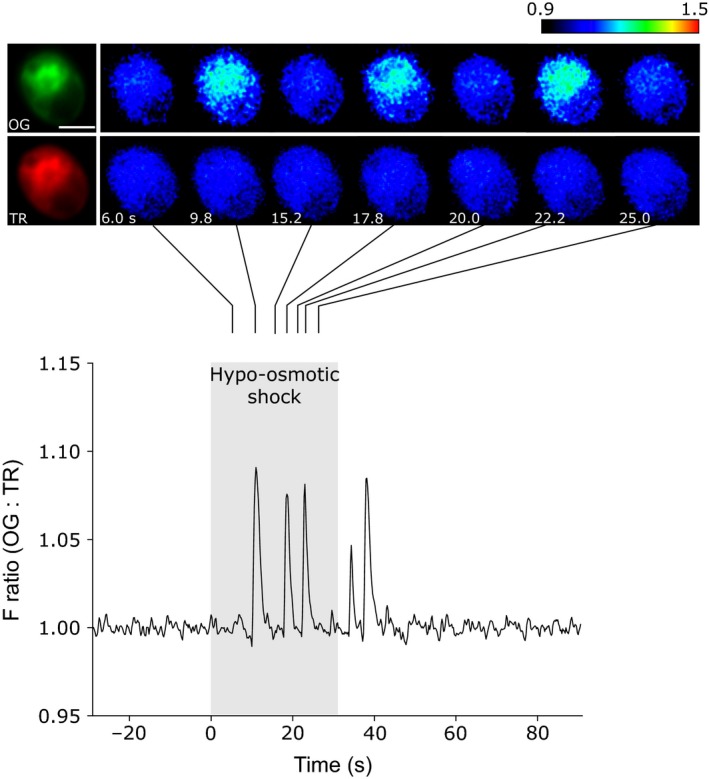
Hypo‐osmotic stress induces repetitive [Ca^2+^]_cyt_ elevations in *Chlamydomonas reinhardtii*. Spatial and temporal characterization of a [Ca^2+^]_cyt_ elevation induced by hypo‐osmotic shock in CC1201 was performed. The graph displays the change in fluorescence ratio (Oregon Green BAPTA dextran : Texas Red dextran (OG : TR)). The pseudocoloured images represent the change in fluorescence (Δ*F*/*F*_0_) in the OG and TR channels. Hypo‐osmotic shock was induced by rapid perfusion of deionized water containing 50 μM CaCl_2_, leading to a series of rapid [Ca^2+^]_cyt_ elevations that appear to be spatially uniform within the cell (300 ms per frame). The cell shown is representative of 31 cells examined. Bar, 5 μm.

Hypo‐osmotic stress causes a rapid influx of water into algal cells and may lead to catastrophic cell bursting if the cell cannot rapidly osmoregulate (Taylor *et al*., 1996). *Chlamydomonas reinhardtii* exists in freshwater and soil environments, which are often hypotonic relative to the cytosol, and uses a contractile vacuole (CV) to expel excess water (Komsic‐Buchmann *et al*., 2014). Preliminary hypo‐osmotic shock experiments using diluted media (50%) failed to induce any [Ca^2+^]_cyt_ elevations in *C. reinhardtii* strain CC1021. However, perfusion of *C. reinhardtii* cells with deionized water (containing 50 μM CaCl_2_) routinely induced a series of repetitive [Ca^2+^]_cyt_ elevations (Fig. 4). We observed at least one [Ca^2+^]_cyt_ elevation in 71% of the cells (*n *=* *31). The mean number of [Ca^2+^]_cyt_ elevations observed within 60 s of hypo‐osmotic shock was 3.93 ± 0.47 and the average duration of each [Ca^2+^]_cyt_ elevation was 1.91 ± 0.50 s (*n *=* *14 cells used for analysis). The mean maximal amplitude of the [Ca^2+^]_cyt_ elevations was 6.08 ± 0.50%, equating to 166 nM [Ca^2+^]_cyt_ (*n *=* *14 cells). The initial [Ca^2+^]_cyt_ elevation occurred 15.93 ± 1.53 s after the stimulus was applied. In contrast to [Ca^2+^]_cyt_ elevations induced by NaCl, the [Ca^2+^]_cyt_ elevations induced by hypo‐osmotic shock did not originate in the apex and appeared to be uniformly distributed throughout the cytosol (Fig. 4). We did not observe [Ca^2+^]_cyt_ elevations in the cell wall‐deficient mutant *cw15* in response to hypo‐osmotic shock (*n *=* *11 cells). To investigate this further, we examined the response to hypo‐osmotic shock in two further *C. reinhardtii* strains. We did not observe any [Ca^2+^]_cyt_ elevations in another cell wall‐deficient strain CC3395 (*n *=* *11 cells), but found that 53.9% of CC125 cells (a walled wild‐type strain) exhibited [Ca^2+^]_cyt_ elevations (*n *=* *13).

The hypo‐osmotic stress applied (deionized water containing 50 μM CaCl_2_) provided a robust and reproducible stimulus that enabled us to study the nature of Ca^2+^ signalling in *C. reinhardtii*. However, the ionic compositions of the pretreatment (CAB) and treatment solutions are not identical and it is possible that changes in pH, Ca^2+^ or other ions also contributed to the observed [Ca^2+^]_cyt_ elevations in addition to the change in osmolarity. To examine this possibility, we applied additional hypo‐osmotic stimuli to *C. reinhardtii* cells. We did not observe [Ca^2+^]_cyt_ elevations when cells acclimated to CAB + 50 mM sorbitol were returned to CAB (*n *=* *7 cells). However, cells acclimated to CAB + 100 mM sucrose gave robust and reproducible [Ca^2+^]_cyt_ elevations when switched to 10% CAB (adjusted to 300 μM Ca^2+^ and pH 7.4) (seven out of 11 cells examined). This suggests that the [Ca^2+^]_cyt_ elevations observed were not attributable to changes in Ca^2+^ or pH. While we cannot rule out a contribution from other ions (e.g. Cl^−^), we conclude that the rapid switch to a solution of very low osmolarity is likely to be the primary contributor to the observed [Ca^2+^]_cyt_ elevations. The initial hypo‐osmotic stimulus (deionized water + 50 μM CaCl_2_) was used for all further experiments.

**Figure 5:**
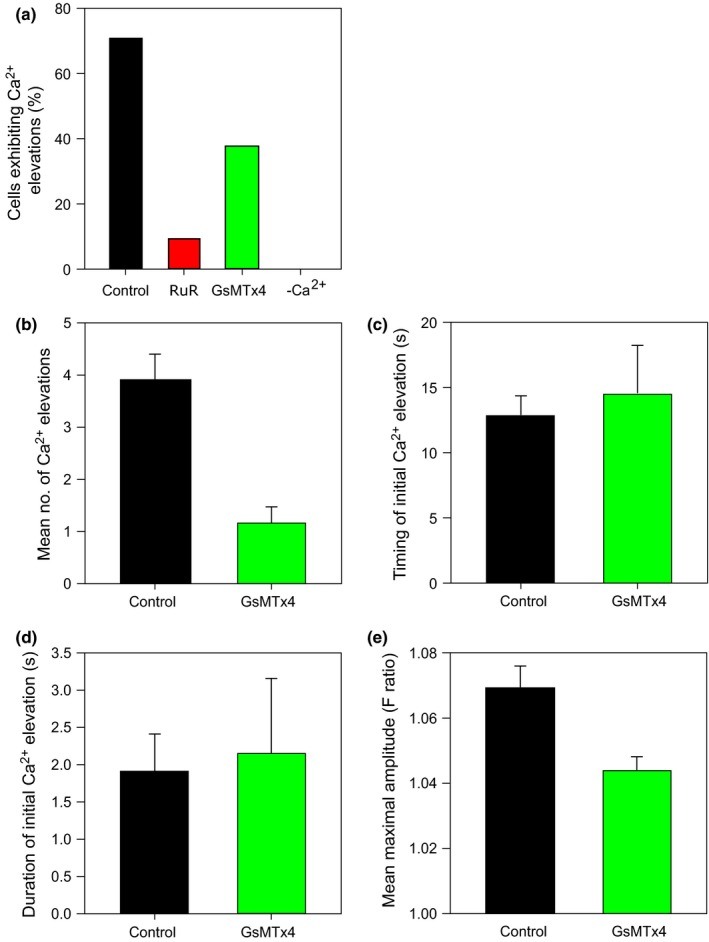
Mechanosensitive ion channels and external Ca^2+^ are required for [Ca^2+^]_cyt_ elevations in response to the hypo‐osmotic stress. (a) The percentage of *Chlamydomonas reinhardtii* CC1021 cells exhibiting at least one [Ca^2+^]_cyt_ elevation following application of hypo‐osmotic shock for 60 s (*n *=* *31 for control cells). Cells were treated either with the mechanosensitive ion channel blockers Ruthenium red (RuR; 10 μM; *n *=* *11) and GsMTx4 (5 μM; *n *=* *16) or by removing external Ca^2+^ (*n *=* *21). (b) Mean number of [Ca^2+^]_cyt_ elevations induced by hypo‐osmotic shock (only cells exhibiting [Ca^2+^]_cyt_ elevations were used for comparison). (c) Timing of initial [Ca^2+^]_cyt_ elevations following hypo‐osmotic shock. (d) Duration of initial [Ca^2+^]_cyt_ elevation. (e) Mean maximal amplitude of initial [Ca^2+^]_cyt_ elevation. Error bars denote SE.

The response to hypo‐osmotic shock in CC1021 cells was dependent on the presence of external Ca^2+^. No cells exhibited [Ca^2+^]_cyt_ elevations within 60 s of the hypo‐osmotic stimulus in the absence of external Ca^2+^ (*n *=* *21) (Fig. 5a). Our results suggest that an influx of external Ca^2+^ is required to trigger [Ca^2+^]_cyt_ elevations in *C. reinhardtii* in response to hypo‐osmotic stress. In many organisms, the activation of mechanosensitive ion channels during cell swelling plays a role in the generation of [Ca^2+^]_cyt_ elevations in response to hypo‐osmotic stress (Taylor *et al*., 1996; Nakayama *et al*., 2012). To test whether mechanosensitive ion channels are required for the initiation of [Ca^2+^]_cyt_ elevations in *C. reinhardtii*, we applied RuR, a nonspecific inhibitor of mechanosensitive channels, and GsMTx4, a specific inhibitor of stretch‐activated ion channels (Bowman *et al*., 2007) (Fig. 5a–e). Ten‐micromolar RuR strongly inhibited [Ca^2+^]_cyt_ elevations (only one out of 11 cells exhibited a single [Ca^2+^]_cyt_ elevation). Five‐micromolar GsMTx4 resulted in a lower proportion of cells exhibiting [Ca^2+^]_cyt_ elevations (38% compared with 71% in the untreated control; *n *=* *16) and these cells only exhibited single rather than repetitive [Ca^2+^]_cyt_ elevations. GsMTx4 did not have any effect on the timing and the duration of the initial [Ca^2+^]_cyt_ elevation.

Ca^2+^‐dependent signalling pathways have been implicated in the regulation of CV activity in several unicellular protists (Allen & Naitoh, 2002; Ladenburger *et al*., 2006). However, we observed no direct correlation between the timing of [Ca^2+^]_cyt_ elevations in *C. reinhardtii* and the water expulsion (systole) events of the CV. Furthermore, we found no evidence to suggest that Ca^2+^ signalling influenced the frequency of systole events during hypo‐osmotic shock. The mean time between systole events was 21.8 ± 1.0 s in control cells and was 17.8 ± 0.7 s during hypo‐osmotic shock (*n *=* *13 cells). In Ca^2+^‐free media, the mean time between systole events was 21.4 ± 1.2 s in untreated control cells and 18.4 ± 0.4 s during hypo‐osmotic shock (*n *=* *18 cells). In cells treated with 10 μM RuR, the mean time between systole events was 20.6 ± 1.2 s before and 20.5 ± 1.1 s during hypo‐osmotic shock (*n *=* *17 cells). The timing of systole events between the treatments was not statistically significant (one‐way ANOVA; Holm–Sidak post hoc test). To examine whether the [Ca^2+^]_cyt_ elevations could be generated in the absence of CV activity, we acclimated *C. reinhardtii* cells to mildly hypertonic medium (CAB + 100 mM sucrose) to minimize CV activity (Komsic‐Buchmann *et al*., 2012). Application of a hypo‐osmotic shock to these cells resulted in significant swelling of the cell body and led to restoration of CV activity within 1–2 min. Multiple [Ca^2+^]_cyt_ elevations were only observed in 50% of these cells (nine out of 18) and these were primarily restricted to the period before the onset of full CV activity (Fig. S4). The remaining cells did not exhibit [Ca^2+^]_cyt_ elevations, but still showed full restoration of CV activity. Thus, the [Ca^2+^]_cyt_ elevations induced by hypo‐osmotic shock are not dependent on CV activity and are not required for the initiation of CV activity.

### [Ca^2+^]_cyt_ elevations induced by hypo‐osmotic shock do not induce deflagellation

*Chlamydomonas reinhardtii* cells have two flagella *c*. 10 μm in length that are used for swimming and gliding motility. Although the two flagella represent only 0.27% of the total cell volume (assuming a cell radius of *c*. 5 μm), they constitute a much larger proportion of the cell surface area (*c*. 7.2%) and may therefore play an important role in osmotic signalling responses. Certain osmotic stimuli have previously been shown to induce deflagellation. For example, application of 50 mM KCl to *Chlamydomonas moeuwsii* caused rapid deflagellation (within 30 s), whereas higher concentrations of KCl caused a much slower deflagellation response (Meijer *et al*., 2002). As deflagellation is directly induced by [Ca^2+^]_cyt_ elevations (Wheeler *et al*., 2008), we examined whether the [Ca^2+^]_cyt_ elevations observed in *C. reinhardtii* during either NaCl or hypo‐osmotic stress triggered the deflagellation response. Using differential interference contrast (DIC) microscopy, we found that salinity stress induced by 300 mM NaCl for 30 s did not result in deflagellation in CC1021 cells (zero out of 43 cells deflagellated). Applying a hypo‐osmotic stress for 30 s also did not induce deflagellation (0 out of 50 cells deflagellated). Examination of cells loaded with Ca^2+^‐responsive dyes confirmed that deflagellation does not occur during [Ca^2+^]_cyt_ elevations induced by hypo‐osmotic shock (seven cells examined where flagella could be visualized). Therefore, the observed [Ca^2+^]_cyt_ elevations induced by these stressors are not linked to the deflagellation process.

### Hypo‐osmotic shock induces compartmentalized Ca^2+^ elevations in flagella

**Figure 6:**
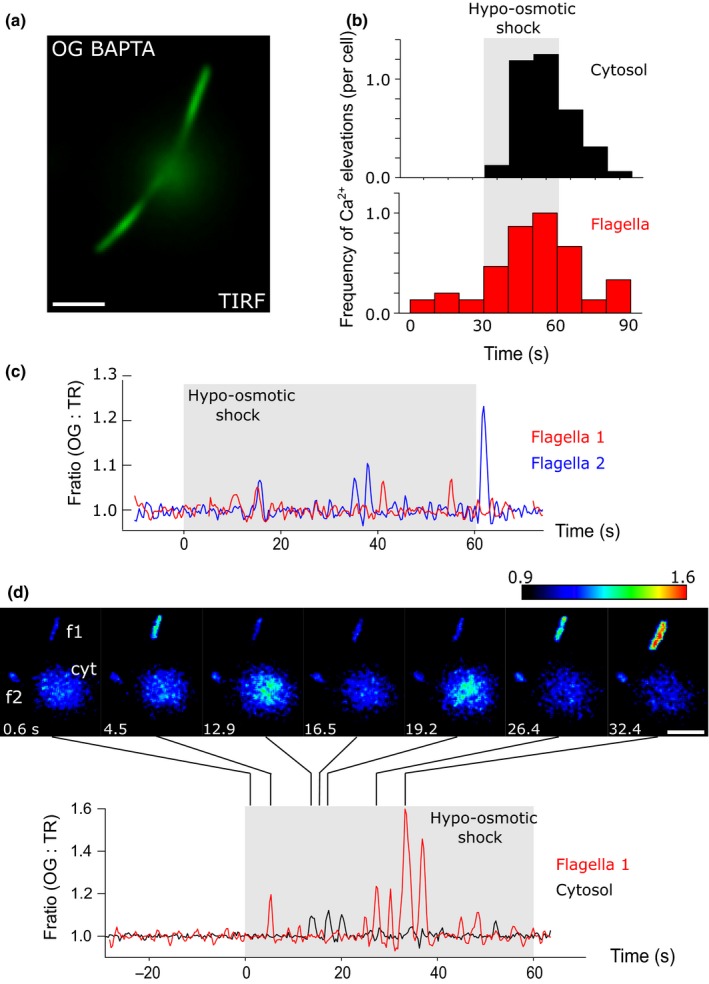
Hypo‐osmotic shock induces repetitive [Ca^2+^]_fla_ elevations in the flagella. (a) Total Internal Reflection Fluorescence (TIRF) microscopy image of a *Chlamydomonas reinhardtii* cell loaded with Oregon Green BAPTA dextran, demonstrating the specific visualization of gliding flagella with minimal interference from the cell body. (b) Frequency histograms indicating the [Ca^2+^] elevations in the cytosol (*n *=* *16) and flagella (*n *=* *15) following hypo‐osmotic shock. Bin size = 10 s. (c) Simultaneous measurement of [Ca^2+^]_fla_ elevations in two flagella from a single cell in response to hypoosmotic shock. Both flagella exhibit repetitive [Ca^2+^]_fla_ elevations, although these do not coincide with each other. The trace is representative of 15 cells examined. (d) Simultaneous measurement of [Ca^2+^] elevations in the flagellum and cytosol of a single cell in response to hypo‐osmotic shock. The hypo‐osmotic shock induces [Ca^2+^] elevations in both compartments, although these do not coincide and appear to be distinct from each other. Note that the amplitude of [Ca^2+^]_fla_ elevations was much greater than those observed in the cytosol following hypo‐osmotic shock. Bars, 5 μm.

We next examined whether hypo‐osmotic shock was able to induce Ca^2+^ elevations in flagella and whether these were related to the observed [Ca^2+^]_cyt_ elevations. We have previously demonstrated that large [Ca^2+^]_cyt_ elevations (induced by the addition of external Ca^2+^) can result in simultaneous [Ca^2+^]_fla_ elevations in both flagella (Wheeler *et al*., 2008). However, it is also clear that [Ca^2+^]_fla_ elevations in individual *C. reinhardtii* flagella can occur independently of the cytosol and the other flagellum (Collingridge *et al*., 2013). Using TIRF microscopy to specifically visualize flagella, we found that hypo‐osmotic shock resulted in multiple rapid [Ca^2+^]_fla_ elevations in 76.7% of flagella examined (*n *=* *26), with an average of 3.06 ± 0.27 Ca^2+^ elevations per flagellum (Fig. 6a,b). Addition of 10 μM RuR completely inhibited the [Ca^2+^]_fla_ elevations caused by hypo‐osmotic shock.

We found that the rapid [Ca^2+^]_fla_ elevations induced by hypo‐osmotic shock in one flagellum did not routinely coincide with [Ca^2+^]_fla_ elevations in the other flagellum, suggesting that each flagellum acts as an independent Ca^2+^ signalling compartment (Fig. 6c). To examine whether [Ca^2+^]_fla_ elevations coincided with [Ca^2+^]_cyt_ elevations, we used a pseudo‐TIRF imaging approach, altering the angle of the excitation light so that we could simultaneously image the flagella and the apical region of the cell body of *C. reinhardtii*. We found that the repetitive [Ca^2+^]_cyt_ and [Ca^2+^]_fla_ elevations induced by hypo‐osmotic shock do not coincide and exhibit no apparent relationship (Fig. 6d) (*n *=* *8 cells). Furthermore, [Ca^2+^]_fla_ elevations were observed in cells that did not demonstrate [Ca^2+^]_cyt_ elevations. The results suggest that the flagella and the cytosol can act as independent Ca^2+^ signalling compartments in response to hypo‐osmotic shock.

**Figure 7:**
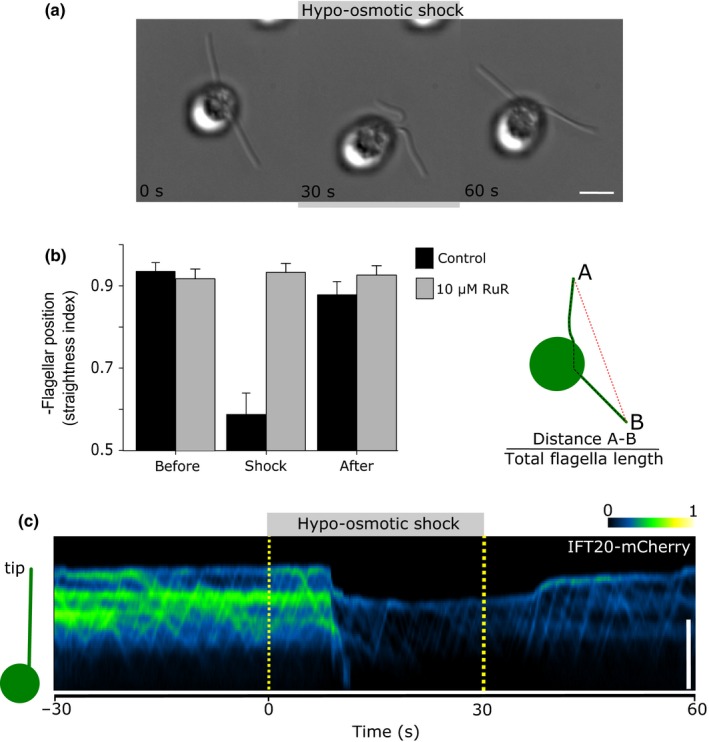
Hypo‐osmotic shock influences flagellar‐mediated gliding motility. (a) Differential interference contrast microscopy images of gliding *Chlamydomonas reinhardtii* CC1021 cells. The images show the position of the flagella in a representative cell immediately before hypo‐osmotic shock (left), after application of hypo‐osmotic shock for 30 s (middle) and after return to normal assay buffer for 30 s (right). (b) Mean straightness of the flagella during hypo‐osmotic shock treatment in control cells (*n *=* *20) and cells treated with 10 μM Ruthenium red (RuR; *n *=* *24). Straightness was defined as the distance measured from tip A to tip B, divided by the total length of the flagellum. (c) Kymograph displaying the movement of intraflagellar transport (IFT) protein IFT20‐mCherry along the flagellum, visualized by Total Internal Reflection Fluorescence (TIRF) microscopy. The anterograde (towards the tip) and retrograde (towards the cell body) movements of IFT particles can be visualized as diagonal lines on the kymograph. Pseudocolour is used to indicate the changes in the fluorescence of IFT20‐mCherry as it accumulates. In the example shown, the IFT particles have accumulated in the stationary flagellum, but these are rapidly dispersed via retrograde IFT following hypo‐osmotic shock. The IFT particles do not accumulate during the duration of the hypo‐osmotic shock, but following the return to normal assay conditions, the IFT particles begin to accumulate and the flagellum begins to glide forward. Bars, 5 μm. Error bars denote SE.

[Ca^2+^]_fla_ elevations modulate flagella‐mediated gliding motility of *C. reinhardtii* cells by regulating the accumulation of IFT particles and their associated molecular motors (Collingridge *et al*., 2013; Shih *et al*., 2013). We found that hypo‐osmotic shock caused gliding CC1021 cells to withdraw their flagella from the 180° configuration, suggesting that the pulling force in each flagellum had been disrupted (Fig. 7a,b). When cells were returned to the original medium, the flagella began to pull forward once more and they rapidly returned to their typical 180° orientation. The effect of hypo‐osmotic shock on gliding motility was inhibited by 10 μM RuR (Fig. 7b). We next examined the impact of hypo‐osmotic shock on the accumulation of IFT particles using a *C. reinhardtii* strain expressing the IFT20‐mCherry reporter fusion (Lechtreck *et al*., 2009). We found that the typical accumulation of IFT particles found in stationary flagella was disrupted following hypo‐osmotic shock, with retrograde transport returning the IFT particles to the cell body (Fig. 7c). In each case, disruption of accumulated IFT particles coincided with the withdrawal of the flagellar tip (*n *=* *27 flagella). When the cells were restored to the assay buffer, the flagellar tips moved forward and IFT particles began to accumulate again. Together, these observations suggest that the [Ca^2+^]_fla_ elevations observed during hypo‐osmotic shock influence flagellar‐mediated gliding motility through their action on the accumulation of IFT particles (Collingridge *et al*., 2013).

## Discussion

### *Chlamydomonas reinhardtii* [Ca^2+^]_cyt_ elevations in response to NaCl stress

Rapid signalling responses to osmotic stimuli are required by nearly all eukaryote cells to prevent swelling or shrinking of the cell which may be potentially damaging. Ca^2+^‐dependent signalling pathways play a critical role in these responses in land plants, and evolution of specific Ca^2+^ signalling properties probably played a major role in the successful colonization of terrestrial environments. Our results demonstrate that Ca^2+^ signalling is likely to be involved in the response of green algae to osmotic stimuli, although the nature of these signals and the manner in which they are generated are likely to differ significantly from those in land plants.

We found that *C. reinhardtii* cells responded to NaCl stress with a single rapid Ca^2+^ elevation. The timing and the amplitude of the signal were dependent on the strength of the stimulus, with larger Ca^2+^ elevations observed at the higher NaCl concentrations. Many aspects of these Ca^2+^ elevations are similar to those observed in vascular plants. Studies using the Ca^2+^‐responsive bioluminescent reporter aequorin revealed that Arabidopsis roots exhibit a single [Ca^2+^]_cyt_ transient when exposed to salt stress (Knight *et al*., 1997). The amplitude of the [Ca^2+^]_cyt_ elevations in Arabidopsis roots was also observed to be greater with increasing NaCl concentration (Tracy *et al*., 2008). However, care must be taken when comparing results obtained using aequorin to single cell imaging, as aequorin reports the mean [Ca^2+^]_cyt_ concentration within a population of cells and therefore will not reflect complex spatial and temporal changes in [Ca^2+^]_cyt_ in an individual cell (Dodd *et al*., 2006). Single‐cell imaging of the moss *Physcomitrella patens* indicated that protonema cells exhibited a single sustained [Ca^2+^]_cyt_ transient in response to 250 mM NaCl (Qudeimat *et al*., 2008), whereas Arabidopsis roots exhibited a biphasic [Ca^2+^]_cyt_ elevation in response to 100 mM NaCl, in which an initial rapid [Ca^2+^]_cyt_ elevation was followed by a much lower secondary [Ca^2+^]_cyt_ elevation (Bonza *et al*., 2013).

Our findings reveal some important distinctions between the NaCl‐induced [Ca^2+^]_cyt_ elevations in single plant cells and those observed in *C. reinhardtii*. The [Ca^2+^]_cyt_ elevations in *C. reinhardtii* are very rapid, rising and returning to basal levels within 5 s, and propagate from the apex of the cell as a fast Ca^2+^ wave. Fast Ca^2+^ waves in animal cells involve the sequential Ca^2+^‐dependent activation of inositol triphosphate receptors (IP_3_Rs) in the endoplasmic reticulum, leading to a spatially propagating [Ca^2+^]_cyt_ elevation. Fast Ca^2+^ waves have not been characterized in detail in individual cells of land plants, with the exception of a UV‐induced fast Ca^2+^ wave in *Physcomitrella patens* caulonema cells (Tucker *et al*., 2005), although spontaneous Ca^2+^ elevations in Arabidopsis guard cells can exhibit spatial propagation (Yang *et al*., 2008). Recently, Choi *et al*. (2014) demonstrated that the application of NaCl to root tips induces an ultrafast intercellular Ca^2+^ wave in Arabidopsis that spreads very rapidly along whole‐plant tissues at 400 μm s^−1^. The mechanism through which this ultrafast Ca^2+^ wave propagates between plant cells is not yet clear, although the speed of the wave was drastically reduced in mutant plants defective in the vacuolar localized two‐pore channel TPC1 (Choi *et al*., 2014). Plant genomes lack homologues of the IP_3_R, which may contribute to the apparent absence of typical fast Ca^2+^ waves in most plant cells (Krinke *et al*., 2007). By contrast, IP_3_R homologues are present in several algae, including *C. reinhardtii* (Wheeler & Brownlee, 2008; Verret *et al*., 2010), where they could potentially contribute to the propagation of a Ca^2+^ wave during salt stress.

Given the requirement for external Ca^2+^, the NaCl‐induced [Ca^2+^]_cyt_ elevations in *C. reinhardtii* are likely to require activation of Ca^2+^‐permeable channels in the plasma membrane, presumably at the cell apex. In Arabidopsis, it has been suggested that Annexin1 (ANN1), a putative plasma membrane Ca^2+^‐permeable channel, is responsible for a Ca^2+^‐dependent Ca^2+^ influx in the presence of 220 mM NaCl (Laohavisit *et al*., 2013). However, annexins have not yet been identified in the genome of *C. reinhardtii* (Jami *et al*., 2012). More recently, another Ca^2+^‐permeable channel, OSCA1, has been identified in plants that contributes to [Ca^2+^]_cyt_ elevations induced by sorbitol (Yuan *et al*., 2014). Several homologues of OSCA1 have been identified in *C. reinhardtii* (Yuan *et al*., 2014; Edel & Kudla, 2015), although these form a distinct clade from the plant OSCA channels and so it remains to be seen whether they can also act as Ca^2+^ channels.

### Repetitive Ca^2+^ elevations in response to hypo‐osmotic shock

*Chlamydomonas reinhardtii* uses a very different osmoregulatory strategy from land plants to cope with hypotonic environments, using CVs to expel excess water instead of using a rigid cell well to generate turgor (Komsic‐Buchmann *et al*., 2014). It is likely that these different osmoregulatory strategies have a major impact on the signalling pathways associated with hypo‐osmotic stress. Many different eukaryotes exhibit [Ca^2+^]_cyt_ elevations in response to hypo‐osmotic stress, including plants, animals, fungi, trypanosomes, brown algae and diatoms (Taylor *et al*., 1996; Falciatore *et al*., 2000; Tatur *et al*., 2007; Hoffmann *et al*., 2009; Nakayama *et al*., 2012). In these organisms, two types of response are commonly observed; either a single [Ca^2+^]_cyt_ elevation or a biphasic response involving Ca^2+^ influx, followed by Ca^2+^ release from intracellular stores. By contrast, the [Ca^2+^]_cyt_ elevations induced by hypo‐osmotic stress in *C. reinhardtii* were highly repetitive in nature. Although repetitive [Ca^2+^]_cyt_ elevations in response to hypo‐osmotic stress have been observed in animal cells, we are not aware of repetitive [Ca^2+^]_cyt_ elevations in photosynthetic organisms, suggesting that this may be a novel feature of the *C. reinhardtii* response.

In many organisms, the generation of [Ca^2+^]_cyt_ elevations in response to hypo‐osmotic shock is linked to cell swelling and the activation of mechanosensitive ion channels (Taylor *et al*., 1996; Hoffmann *et al*., 2009; Nakayama *et al*., 2012). Previous researchers have demonstrated that mechanical stimulation of the cell body of *C. reinhardtii* results in inward Ca^2+^ currents that are sensitive to Gd^3+^, a nonspecific blocker of mechanosensitive ion channels (Yoshimura, 1998). It seems likely that cell swelling in *C. reinhardtii* caused by hypo‐osmotic shock activates mechanosensitive ion channels in the plasma membrane, leading to an influx of Ca^2+^. Interactions between the cell wall and the plasma membrane may contribute to the response to hypo‐osmotic stress, as we did not observe [Ca^2+^]_cyt_ elevations in cell wall‐deficient mutants, although it has been suggested that the absence of a cell wall in *C. reinhardtii* may alter the osmolarity of the cytosol, which could also influence the response to a hypo‐osmotic stimulus (Hoffmann & Beck, 2005). In animal cells, the [Ca^2+^]_cyt_ elevations induced by hypo‐osmotic shock activate Ca^2+^‐activated K^+^ channels and induce solute release, preventing cell bursting and contributing to the regulatory volume decrease (Hoffmann *et al*., 2009). Ca^2+^ plays a similar role in osmotic adjustment in brown algae, with disruption of Ca^2+^ signalling leading to cell bursting in *Fucus* embryos (Taylor *et al*., 1996). As the [Ca^2+^]_cyt_ elevations generated during hypo‐osmotic shock in *C. reinhardtii* do not appear to contribute to the regulation of CV function, it is likely that they play a role in rapid osmotic adjustment.

Recent progress has begun to identify the molecular mechanisms involved in hypo‐osmotic signalling in other eukaryotes. In fission yeast, two endoplasmic reticulum‐localized mechanosensitive ion channels belonging to the MscS‐like (MSL) family (Msy1 and Msy2) contribute to Ca^2+^ elevations generated during hypo‐osmotic shock (Nakayama *et al*., 2012). Although plants contain a large family of MSL channels, their role in generating [Ca^2+^]_cyt_ elevations is not clear and another group of mechanosensitive ion channels, the Mid1‐complementing activity (MCA) proteins, probably contribute to Ca^2+^ influx during hypo‐osmotic shock (Nakagawa *et al*., 2007; Haswell *et al*., 2008). *Chlamydomonas reinhardtii* does not possess MCA proteins and, although it possesses three MSL proteins, these may have other roles in the cell, as characterization of MSC1 revealed it to be a chloroplast‐localized Cl^−^ channel (Nakagawa *et al*., 2007). *Chlamydomonas reinhardtii* does, however, contain an expanded family of TRP channels (Wheeler & Brownlee, 2008; Fujiu *et al*., 2011; Arias‐Darraz *et al*., 2015) and this class of channel plays an important role in osmotic signalling in mammals (Hoffmann *et al*., 2009).

### The role of the cell wall in osmotic Ca^2+^ signalling

We observed distinct Ca^2+^ signalling responses of walled and wall‐less strains to osmotic stimuli. The wild‐type walled strain CC1021 was much less sensitive to NaCl than the wall‐less strain *cw15*, but conversely the wall‐less strains did not exhibit Ca^2+^ elevations in response to hypo‐osmotic stress. These observations suggest that the cell wall may contribute to osmotic signalling, although the underlying mechanisms are not yet clear. Interactions between the plasma membrane and the cell wall have been suggested to contribute to mechanical stimuli during both hypo‐ and hyperosmotic stress in *Fucus* rhizoids (Taylor *et al*., 1996). Interactions with the cell wall could contribute to the [Ca^2+^]_cyt_ elevations generated by hypo‐osmotic stimuli in *C. reinhardtii*, as [Ca^2+^]_cyt_ elevations were not observed in wall‐less strains, although they did respond to NaCl. The differing sensitivities of wall‐less mutants to osmotic stimuli may also arise from the physiological consequences of the absence of a cell wall. Walled and wall‐less strains exhibit similar levels of CV activity, suggesting that the cell wall only has a minor impact on water influx (Luykx *et al*., 1997; Komsic‐Buchmann *et al*., 2012). Cytosolic osmolarity was estimated to be slightly greater in wall‐less strains (Luykx *et al*., 1997; Komsic‐Buchmann *et al*., 2012), which could influence the response to osmotic stimuli, although this does not appear to be the case in the response to hypo‐osmotic stress, as we would expect the wall‐less strains to be more sensitive rather than less sensitive.

### Flagella act as independent Ca^2+^ signalling compartments

Previous studies have found that *C. reinhardtii* flagella are mechanosensitive. Mechanical stimulation of *C. reinhardtii* flagella using a suction pipette results in a series of repetitive inward Ca^2+^ currents (Yoshimura, 1996) and swimming cells exhibit a mechano‐shock response (a brief period of backwards swimming), which is mediated by a flagellar‐localized TRP channel (TRP11) (Fujiu *et al*., 2011). Similarly, applying a stretching force to a flagellum attached to a substrate results in repetitive flagella Ca^2+^ elevations (Collingridge *et al*., 2013). It seems likely that water influx into the flagellum during hypo‐osmotic shock may cause swelling, which could activate these mechanosensitive Ca^2+^ responses. The dynamic [Ca^2+^]_fla_ elevations observed during hypo‐osmotic shock may therefore contribute to localized osmotic adjustment in flagella, for example through the activation of Ca^2+^‐activated K^+^ channels. Although we found that hypo‐osmotic shock influenced gliding motility, it remains unclear whether this is simply a consequence of raising [Ca^2+^]_fla_ in this organelle. [Ca^2+^]_fla_ elevations contribute to the detachment of the flagella from the substrate by causing the dissociation of IFT particles from the adherent flagella membrane glycoproteins (Collingridge *et al*., 2013). During hypo‐osmotic shock, this response may enable the previously immotile cell to detach from the substrate and move to more favourable areas. A potent inhibitor of Ca^2+^‐activated K^+^ channels (ciliabrevin) was found to cause flagellar shortening and deflagellation in *C. reinhardtii* (Engel *et al*., 2011) and it will be interesting to determine whether this phenotype arises from an inability of flagella to osmoregulate.

The vastly different morphologies of the flagella and the cell body suggest that they will have differing sensitivities to osmotic stress. The ability of the cell to regulate Ca^2+^ independently between the flagella and the cytosol enables a localized cellular response dependent on the nature of the stimulus. Although our results indicate localized Ca^2+^ signalling in flagella during osmotic shock in *C. reinhardtii*, other flagellar signalling processes are strictly dependent on the cell body and include the well‐characterized photoresponses in which light sensing by the eyespot leads to the activation of flagellar‐localized Ca^2+^ channels (Harz & Hegemann, 1991). *Chlamydomonas reinhardtii* flagella therefore represent highly dynamic excitable signalling compartments that can act either independently of or in combination with the cell body. Recent findings suggest that the motile cilia in mammalian cells are much less independent of the cell body, as ciliary Ca^2+^ was found to be highly dependent on [Ca^2+^]_cyt_ (Doerner *et al*., 2015). Moreover, the nonmotile primary cilia of mammalian cells differ further as they have an elevated resting [Ca^2+^] (750 nM) and do not exhibit dynamic changes in [Ca^2+^] (Delling *et al*., 2013). These observations suggest that there is considerable diversity between organisms in the mechanisms underlying ciliary Ca^2+^ elevations and their interactions with the cytosol.

There is considerable interest in the role of cilia as cellular sensors and their contribution to the generation of [Ca^2+^]_cyt_ elevations. The potential role of ciliary Ca^2+^ signalling in mechanosensation by the nonmotile mammalian primary cilia has been extensively explored (Nauli *et al*., 2003). However, recent findings demonstrate that mechanical stimulation of primary cilia in kidney epithelial cells and the developing embryo and of the kinocilia of the ear do not induce ciliary Ca^2+^ elevations (Delling *et al*., 2016). By contrast, there is considerable evidence supporting a role for ciliary Ca^2+^ signalling in unicellular protists following mechanical stimulation (Eckert & Brehm, 1979; Yoshimura, 1996; Collingridge *et al*., 2013). Although *C. reinhardtii* flagella do appear to be directly mechanosensitive, in *Paramecium caudatum* mechanosensation is initiated by the cell body rather than the cilium itself (Ogura & Machemer, 1980). Therefore, in many cases the hypothesis that cilia act as cellular sensors and contribute to the generation of [Ca^2+^]_cyt_ elevations must be viewed with caution.

We found no evidence to suggest that the [Ca^2+^]_fla_ elevations in *C. reinhardtii* contribute to [Ca^2+^]_cyt_ elevations. The [Ca^2+^]_fla_ elevations induced by hypo‐osmotic shock are restricted to the flagella, although the possibility remains that these signalling processes could relay information to the cell body through other mechanisms, for example through the generation of mobile second messengers, which could allow detection of potentially damaging osmotic conditions before they influence the cell body directly. An osmosensor role has also been proposed for primary cilia of cholangiocytes in the mammalian biliary duct (Gradilone *et al*., 2007), although clearly the underlying mechanisms may differ. Further detailed examination of the interactions between [Ca^2+^]_cyt_ and [Ca^2+^]_fla_ elevations will be required to test whether *Chlamydomonas* flagella can also function as osmosensors for the cell body.

### Conservation of Ca^2+^ signalling in the green lineage

Our results demonstrate a role for Ca^2+^ signalling the perception of environmental stimuli in the green algae. The central role for Ca^2+^ in osmotic stress signalling appears to be conserved between the major lineages in the Viridiplantae, and this role is also conserved in many other eukaryotes. However, the nature of the [Ca^2+^]_cyt_ elevations in *C. reinhardtii* is distinct from those observed in vascular plants, exemplified by the fast Ca^2+^ wave induced by salt stress or the multiple rapid [Ca^2+^]_cyt_ elevations in response to hypo‐osmotic stress. These differences are probably attributable to a combination of the different Ca^2+^ signalling toolkits found in plants and green algae and wider differences in their morphologies and physiologies. Understanding these differences will not only provide much needed information on stress signalling in green algae but also help us understand the evolution of the signalling processes that enabled the colonization of terrestrial environments by land plants.

## Author contributions

P.B., S.S. and G.L.W. performed the research and analysed the data. G.L.W., J.K.P. and C.B. designed the study. P.B., S.S., J.K.P., C.B. and G.L.W. wrote the manuscript.

## Supporting information

Please note: Wiley Blackwell are not responsible for the content or functionality of any Supporting Information supplied by the authors. Any queries (other than missing material) should be directed to the *New Phytologist* Central Office.

**Fig. S1** Processing of data during Ca^2+^ imaging.
**Fig. S2** *In vitro* calibration of Oregon Green BAPTA dextran.
**Fig. S3** Spontaneous [Ca^2+^]_cyt_ elevations induced by 10 mM external Ca^2+^.
**Fig. S4** [Ca^2+^]_cyt_ elevations induced by hypo‐osmotic stress occur in the absence of contractile vacuole activity.

**Video S1** A Ca^2+^ wave in *Chlamydomonas reinhardtii* induced by NaCl shock.
