# Supplementary material for: Spatial and temporal specificity of Ca2+ signalling in Chlamydomonas reinhardtii in response to osmotic stress
Source: New Phytol. 2016 Aug 12;212(4):920–33. doi: 10.1111/nph.14128 (PMC5111745; doi:10.1111/nph.14128)
Supplement: Supplementary file 1 — Fig. S1 Processing of data during Ca2+ imaging. Fig. S2 In vitro calibration of Oregon Green BAPTA dextran. Fig. S3 Spontaneous [Ca2+]cyt elevations induced by 10 mM external Ca2+. Fig. S4 [Ca2+]cyt elevations induced by hypo‐osmotic stress occur in the absence of contractile vacuole activity. [file NPH-212-920-s001.pdf]

New Phytologist

***New Phytologist* Supporting Information Figs S1–S4**

Article title: Spatial and temporal specificity of Ca^{2+} signalling in *Chlamydomonas reinhardtii* in response to osmotic stress

Authors: Peter Bickerton, Simone Sello, Colin Brownlee, Jon K. Pittman and Glen L. Wheeler

Article acceptance date: 25 June 2016

The following Supporting Information is available for this article:

Fig. S1 Processing of data during Ca^{2+} imaging.

Fig. S2 *In vitro* calibration of Oregon Green BAPTA dextran.

Fig. S3 Spontaneous $[Ca^{2+}]_{cyt}$ elevations induced by 10 mM external Ca^{2+}.

Fig. S4 $[Ca^{2+}]_{cyt}$ elevations induced by hypoosmotic stress occur in the absence of contractile vacuole activity.

Video S1 A Ca^{2+} wave in *Chlamydomonas* induced by NaCl shock (separate mp4 file).

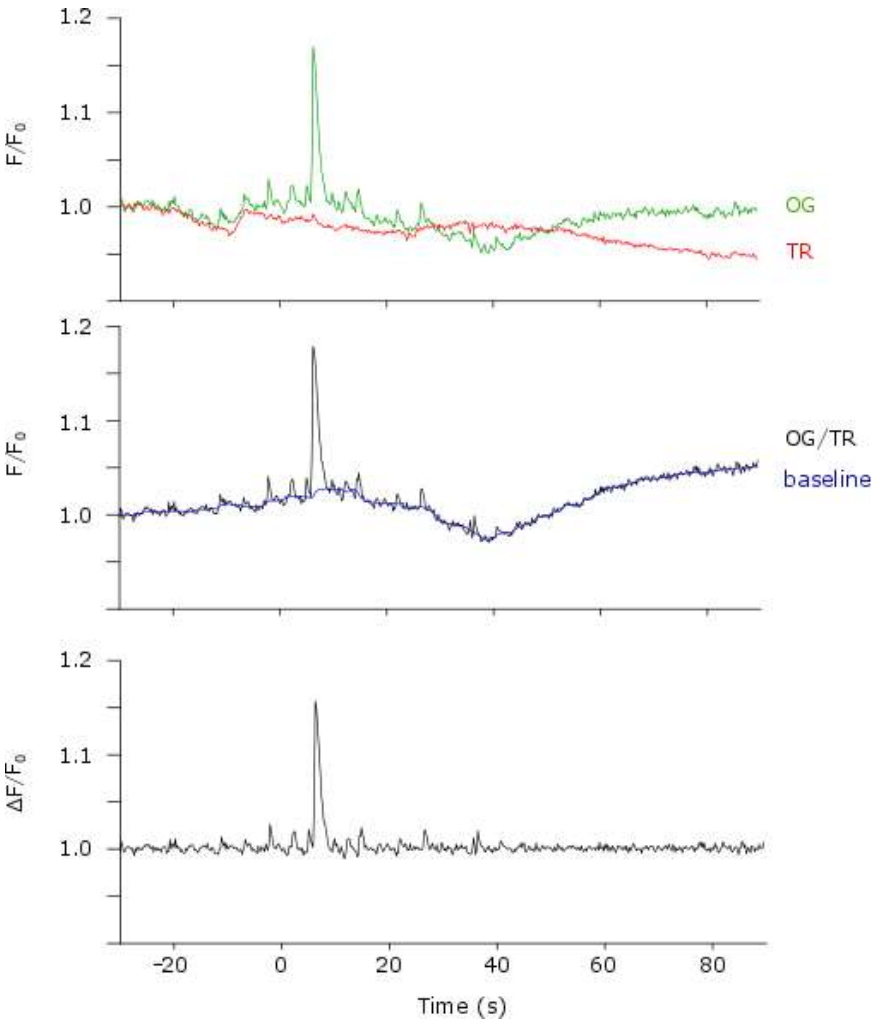

Fig. S1 Data processing during Ca^{2+} imaging. Changes in $[Ca^{2+}]_{cyt}$ were identified by calculating the fluorescence intensity ratio between the Ca2+-responsive Oregon Green BAPTA dextran (OG) and Texas Red dextran (TR) reference dye. A defined region of interest within each *Chlamydomonas* cell was used to generate a trace of mean fluorescence intensity for each dye. The ratio of OG/TR fluorescence was calculated and smoothed using a Savitsky–Golay filter. A baseline trace was then created using an asymmetric least squares smoothing baseline algorithm. The OG/TR trace was divided by the baseline trace to determine the relative change in fluorescence.

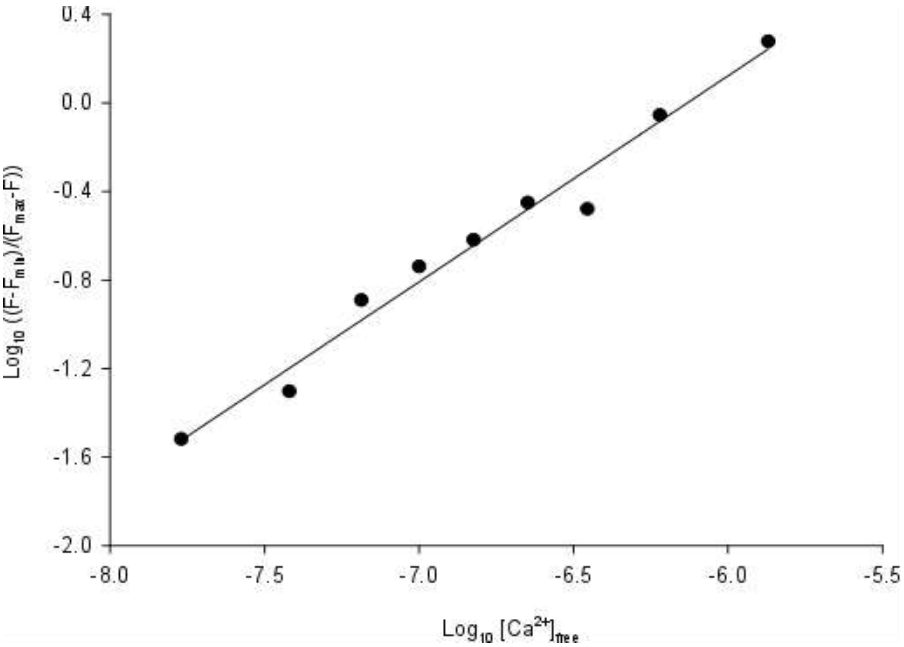

**OG/TR (% baseline)**	**$[Ca^{2+}]_{cyt}$ (nM)**
100	100
110	194
120	297
130	429
140	603
150	838
160	1172

Fig. S2 *In vitro* calibration of Oregon Green BAPTA dextran. The graph (left panel) displays the results of an *in vitro* calibration of the Ca^{2+}-responsive dye Oregon Green BAPTA dextran using the epifluorescent imaging conditions used to image dye-loaded *Chlamydomonas* cells. The calibration can be used to estimate the $[Ca^{2+}]_{cyt}$ following an increase in fluorescence (right panel), assuming a resting $[Ca^{2+}]_{cyt}$ of 100 nM.

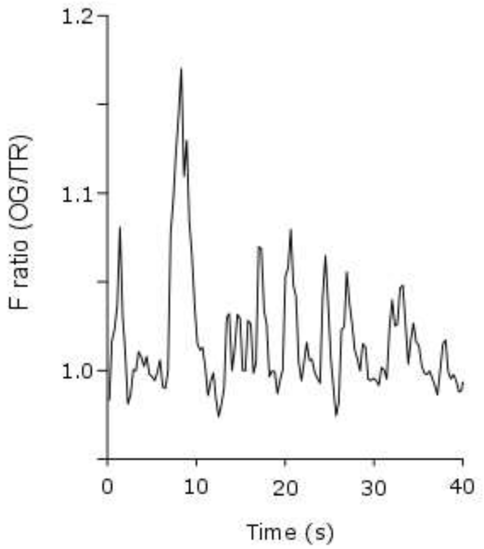

Fig. S3 Spontaneous $[Ca^{2+}]_{cyt}$ elevations induced by 10 mM external Ca^{2+}. *Chlamydomonas* CC1021 cells acclimated to 10 mM external Ca^{2+} for 1 h exhibit a high frequency of spontaneous $[Ca^{2+}]_{cyt}$ elevations. The trace shows an example of spontaneous $[Ca^{2+}]_{cyt}$ elevations exhibited by an unstimulated cell. All cells examined exhibited spontaneous $[Ca^{2+}]_{cyt}$ elevations (n = 5).

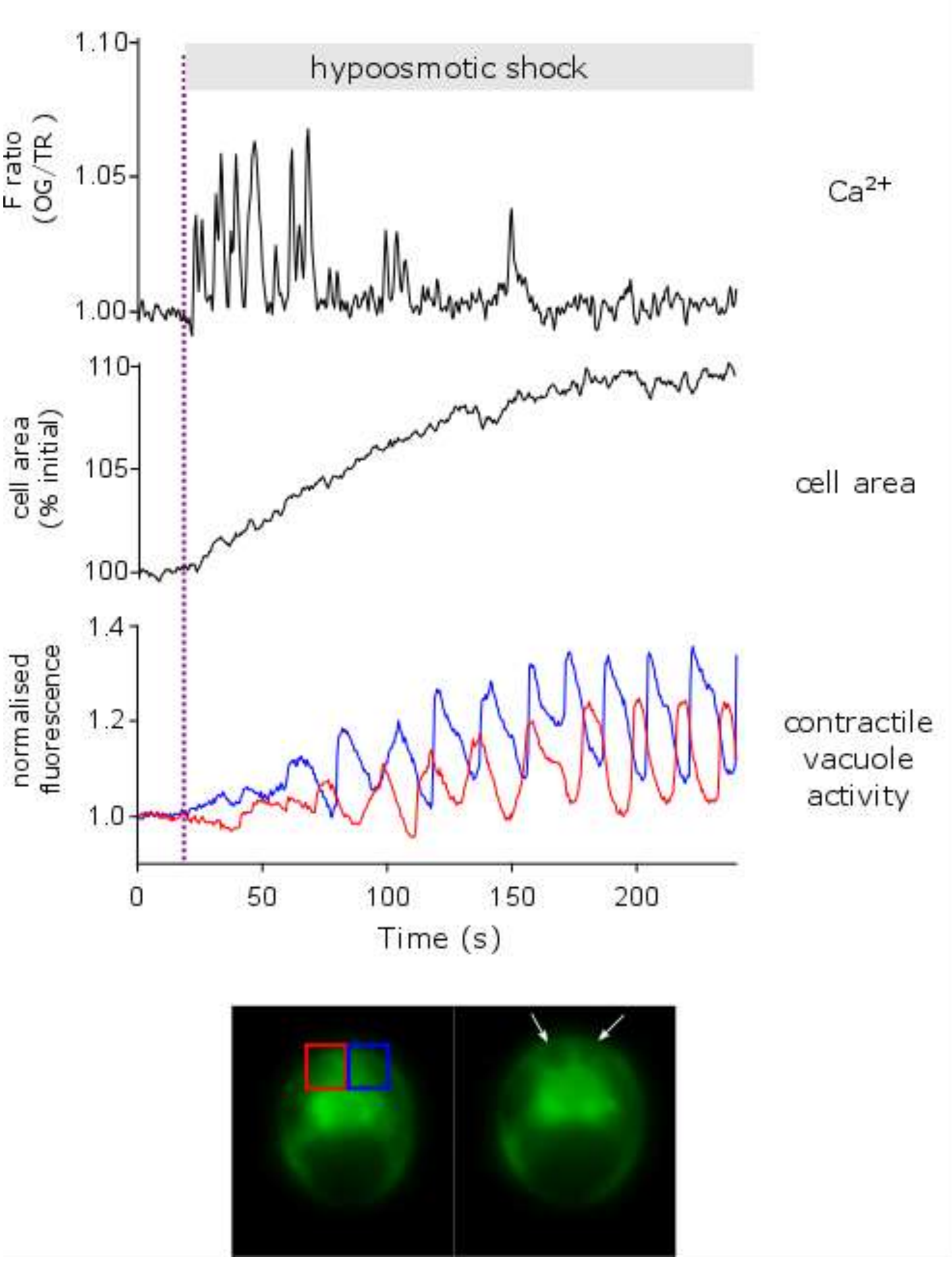

Fig. S4 $[Ca^{2+}]_{cyt}$ elevations induced by hypoosmotic stress occur in the absence of contractile vacuole activity. CC1021 cells were incubated in a hyperosmotic environment (CAB + 100 mM sucrose) to inhibit contractile vacuole (CV) activity. The traces show the changes in $[Ca^{2+}]_{cyt}$, cell size and contractile vacuole activity following the application of a hypoosmotic shock. In the example shown, the hypoosmotic shock causes multiple Ca^{2+} elevations and a pronounced increased in the cell size (measured as cell area). The contractile vacuoles (arrowed) are inactive in the hyperosmotic environment, but regain full activity within 180 s of the hypoosmotic shock.

CV activity was measured by monitoring Texas Red fluorescence in two regions of interest in the apical region of the cell (red and blue squares shown on images below).

Video S1 A Ca^{2+} wave in *Chlamydomonas* induced by NaCl shock. The video shows a $[Ca^{2+}]_{cyt}$ elevation in a *cw15* cell following the addition of 120 mM NaCl. The pseudocoloured images indicate changes in $[Ca^{2+}]_{cyt}$. The apical region of the cell is positioned at the top right of the image. Following the addition of 120 mM NaCl, a $[Ca^{2+}]_{cyt}$ elevation initiates in the apical region and rapidly propagates to the rest of the cell. Images were collected every 300 ms and are displayed at a rate of 10 frames s^{-1}. For further details see Fig. 3.
